# Supplementary material for: MetaRibo-Seq measures translation in microbiomes
Source: Nat Commun. 2020 Jun 29;11:3268. doi: 10.1038/s41467-020-17081-z (PMC7324362; doi:10.1038/s41467-020-17081-z)
Supplement: Supplementary file 10 — Supplementary Data 7 [file 41467_2020_17081_MOESM10_ESM.zip › File2/Confidence_High_Taxonomy/156270_out.krona.html]

Javascript must be enabled to view this page.

members
magnitude
magnitudeUnassigned
count
unassigned
taxon
rank

156270\_out

201

superkingdom
201
2

1239
200
phylum

200
909932
class

1843489
99
order

31977
99
family

species

SRS017497\_contig\_number\_contig-100\_676.676SRS146924\_contig\_number\_219SRS149783\_contig\_number\_138
1384082
3

156454
2
genus

156456

SRS013950\_contig\_number\_contig-100\_32.32SRS014691\_contig\_number\_contig-100\_20672.112023
2
species

10
genus

SRS011243\_contig\_number\_contig-100\_3626.3626SRS014684\_contig\_number\_contig-100\_380.380.380SRS017808\_contig\_number\_contig-100\_429.32968.32968SRS023617\_contig\_number\_11476
29465
4

1110546

SRS075404\_contig\_number\_11276
1
species

2
2292078

SRS015174\_contig\_number\_contig-100\_427.427SRS1055097\_contig\_number\_21
species

species
1

SRS078738\_contig\_number\_contig-100\_724.90406
450749

species
39778

SRS148973\_contig\_number\_10210
1

species
1203593

SRS047210\_contig\_number\_125
1

84
906
genus

species
1232428

SRS015264\_contig\_number\_686SRS065176\_contig\_number\_248SRS1041091\_contig\_number\_155SRS893373\_contig\_number\_843
4

3

SRS103987\_contig\_number\_177SRS143466\_contig\_number\_contig-100\_16.17SRS143466\_contig\_number\_contig-100\_96.52764
1603888
species

species

SRS015431\_contig\_number\_14324SRS043667\_contig\_number\_36SRS053356\_contig\_number\_50SRS1041039\_contig\_number\_6462
907
4

species
187326

SRS013234\_contig\_number\_contig-100\_56.56.56SRS013234\_contig\_number\_contig-100\_1543.31700.31700SRS013651\_contig\_number\_1016SRS014470\_contig\_number\_2716SRS014689\_contig\_number\_contig-100\_29.29SRS014935\_contig\_number\_2004SRS015057\_contig\_number\_contig-100\_146.30056SRS015434\_contig\_number\_5729SRS015941\_contig\_number\_1854SRS016133\_contig\_number\_1817SRS016456\_contig\_number\_903SRS016647\_contig\_number\_contig-100\_1059.1060SRS016740\_contig\_number\_627SRS017120\_contig\_number\_273SRS018610\_contig\_number\_91SRS018875\_contig\_number\_228SRS019022\_contig\_number\_contig-100\_3948.32849SRS019026\_contig\_number\_25SRS019071\_contig\_number\_10881SRS019270\_contig\_number\_54SRS020334\_contig\_number\_161SRS020571\_contig\_number\_contig-100\_421.35199SRS022621\_contig\_number\_contig-100\_534.35765SRS024277\_contig\_number\_11585SRS024498\_contig\_number\_76SRS024580\_contig\_number\_contig-100\_398.29538.29538SRS024637\_contig\_number\_137SRS042131\_contig\_number\_contig-100\_6935.41190SRS049448\_contig\_number\_contig-100\_946.34754SRS052518\_contig\_number\_11151SRS056323\_contig\_number\_118SRS056327\_contig\_number\_36SRS056896\_contig\_number\_contig-100\_15074.61032SRS076868\_contig\_number\_279SRS077092\_contig\_number\_contig-100\_6173.36303SRS077327\_contig\_number\_contig-100\_7949.7950SRS077633\_contig\_number\_166SRS098065\_contig\_number\_170SRS098612\_contig\_number\_630SRS098818\_contig\_number\_contig-100\_15813.15813SRS100027\_contig\_number\_274SRS102808\_contig\_number\_12748SRS103971\_contig\_number\_contig-100\_3122.66333SRS104039\_contig\_number\_contig-100\_6592.42440SRS1041170\_contig\_number\_2319SRS1054881\_contig\_number\_4501SRS1055071\_contig\_number\_49SRS142531\_contig\_number\_3531SRS142680\_contig\_number\_2742SRS143231\_contig\_number\_contig-100\_24.25SRS143310\_contig\_number\_16405SRS143450\_contig\_number\_2096SRS143529\_contig\_number\_3523SRS143850\_contig\_number\_11297SRS143879\_contig\_number\_7912SRS144194\_contig\_number\_79SRS144560\_contig\_number\_23998SRS144609\_contig\_number\_3081SRS144644\_contig\_number\_3447SRS146894\_contig\_number\_2323SRS147094\_contig\_number\_12534SRS147712\_contig\_number\_178SRS148303\_contig\_number\_3067SRS148408\_contig\_number\_52SRS148676\_contig\_number\_36SRS148973\_contig\_number\_58SRS149671\_contig\_number\_15168SRS149690\_contig\_number\_139SRS150032\_contig\_number\_contig-100\_10428.10429SRS893289\_contig\_number\_contig-100\_2758.2759SRS893290\_contig\_number\_206SRS893291\_contig\_number\_64SRS893363\_contig\_number\_103
73

order
101
909929

1843491
101
family

2
970

SRS016815\_contig\_number\_contig-100\_408.27423SRS1041114\_contig\_number\_contig-100\_4715.4716
genus
100

species
1
1944634

SRS147120\_contig\_number\_424


SRS023964\_contig\_number\_45SRS1055037\_contig\_number\_13988SRS144124\_contig\_number\_contig-100\_857.82689
999423
3
species

2
1884263

SRS020226\_contig\_number\_contig-100\_2.32954SRS052061\_contig\_number\_1596
species

4
712532

SRS052095\_contig\_number\_4416SRS104320\_contig\_number\_13561SRS1055073\_contig\_number\_contig-100\_405.32411SRS148205\_contig\_number\_2844
species


SRS018739\_contig\_number\_3063SRS047824\_contig\_number\_47SRS063485\_contig\_number\_11809SRS143068\_contig\_number\_123
69823
4
species


SRS013723\_contig\_number\_7SRS016043\_contig\_number\_8323SRS016746\_contig\_number\_26498SRS018337\_contig\_number\_7290SRS019276\_contig\_number\_10SRS021960\_contig\_number\_7770SRS023938\_contig\_number\_11882SRS024021\_contig\_number\_3664SRS024355\_contig\_number\_12067SRS047126\_contig\_number\_276SRS104521\_contig\_number\_8361SRS144237\_contig\_number\_15281SRS893335\_contig\_number\_11120
135083
13
species

50
712538

SRS015038\_contig\_number\_contig-100\_565.565SRS018610\_contig\_number\_1929SRS019170\_contig\_number\_2549SRS019327\_contig\_number\_5874SRS019488\_contig\_number\_680SRS019894\_contig\_number\_14881SRS021954\_contig\_number\_11972SRS024138\_contig\_number\_11512SRS024498\_contig\_number\_459SRS044373\_contig\_number\_13911SRS047219\_contig\_number\_48SRS051791\_contig\_number\_555SRS053854\_contig\_number\_235SRS062540\_contig\_number\_2645SRS062714\_contig\_number\_11395SRS063193\_contig\_number\_15613SRS075347\_contig\_number\_13243SRS075404\_contig\_number\_1180SRS075813\_contig\_number\_153SRS077216\_contig\_number\_330SRS101382\_contig\_number\_15738SRS104275\_contig\_number\_contig-100\_627.627SRS104287\_contig\_number\_333SRS104384\_contig\_number\_2210SRS1055063\_contig\_number\_310SRS142527\_contig\_number\_683SRS142896\_contig\_number\_1251SRS142896\_contig\_number\_contig-100\_1661.30606SRS143062\_contig\_number\_122SRS143310\_contig\_number\_1061SRS143529\_contig\_number\_9995SRS143726\_contig\_number\_2271SRS144194\_contig\_number\_5438SRS144300\_contig\_number\_6169SRS146767\_contig\_number\_1343SRS147178\_contig\_number\_contig-100\_1017.1018SRS147712\_contig\_number\_119SRS148877\_contig\_number\_2133SRS149231\_contig\_number\_904SRS149280\_contig\_number\_1952SRS149328\_contig\_number\_430SRS149486\_contig\_number\_130SRS149671\_contig\_number\_2689SRS149961\_contig\_number\_2026SRS893260\_contig\_number\_1787SRS893286\_contig\_number\_contig-100\_143.33319SRS893321\_contig\_number\_332SRS893352\_contig\_number\_contig-100\_52.53SRS893359\_contig\_number\_1657SRS893379\_contig\_number\_7568
species


SRS013927\_contig\_number\_2574SRS016002\_contig\_number\_352SRS045715\_contig\_number\_1910SRS056461\_contig\_number\_8688SRS063603\_contig\_number\_6SRS1055039\_contig\_number\_contig-100\_7039.38759SRS143996\_contig\_number\_2488SRS144380\_contig\_number\_27999
135082
8
species

4
713030

SRS018820\_contig\_number\_115SRS024318\_contig\_number\_21SRS144512\_contig\_number\_4544SRS893348\_contig\_number\_170
species

species
671224

SRS012285\_contig\_number\_11914SRS019798\_contig\_number\_9283SRS024447\_contig\_number\_3238SRS044448\_contig\_number\_21SRS049268\_contig\_number\_12SRS077390\_contig\_number\_3918SRS097907\_contig\_number\_12658SRS104921\_contig\_number\_contig-100\_1250.61979SRS149190\_contig\_number\_2585
9

genus
52225
1

species
1

SRS015272\_contig\_number\_193
1321780

201174
1
phylum

1
1760
class

order
2037
1

family
1
2049

1654
1
genus

species

SRS045638\_contig\_number\_contig-100\_143.36503
55565
1
